# Preserved Suppression of Salient Irrelevant Stimuli During Visual Search in Age-Associated Memory Impairment

**DOI:** 10.3389/fpsyg.2015.02033

**Published:** 2016-01-12

**Authors:** Laura Lorenzo-López, Ana Maseda, Ana Buján, Carmen de Labra, Elena Amenedo, José C. Millán-Calenti

**Affiliations:** ^1^Gerontology Research Group, Department of Medicine, Faculty of Health Sciences, Universidade da CoruñaA Coruña, Spain; ^2^Research, Development and Innovation Department, Gerontological Complex La Milagrosa, Provincial Association of Pensioners and Retired People (UDP) from A CoruñaA Coruña, Spain; ^3^Department of Clinical Psychology and Psychobiology, University of Santiago de CompostelaSantiago de Compostela, Spain

**Keywords:** AAMI, automatic attentional capture, ERPs, N2pc, visual search

## Abstract

Previous studies have suggested that older adults with age-associated memory impairment (AAMI) may show a significant decline in attentional resource capacity and inhibitory processes in addition to memory impairment. In the present paper, the potential attentional capture by task-irrelevant stimuli was examined in older adults with AAMI compared to healthy older adults using scalp-recorded event-related brain potentials (ERPs). ERPs were recorded during the execution of a visual search task, in which the participants had to detect the presence of a target stimulus that differed from distractors by orientation. To explore the automatic attentional capture phenomenon, an irrelevant distractor stimulus defined by a different feature (color) was also presented without previous knowledge of the participants. A consistent N2pc, an electrophysiological indicator of attentional deployment, was present for target stimuli but not for task-irrelevant color stimuli, suggesting that these irrelevant distractors did not attract attention in AAMI older adults. Furthermore, the N2pc for targets was significantly delayed in AAMI patients compared to healthy older controls. Together, these findings suggest a specific impairment of the attentional selection process of relevant target stimuli in these individuals and indicate that the mechanism of top-down suppression of entirely task-irrelevant stimuli is preserved, at least when the target and the irrelevant stimuli are perceptually very different.

## Introduction

Memory impairment is common in people over the age of 65. When this memory decline has no underlying medical cause, it is known as age-associated memory impairment (AAMI), which has been considered as a non-progressive normal decline due to aging (Crook et al., 1986; Youngjohn and Crook, 1993; Hänninen et al., 1995). Information on the prevalence rates of AAMI is scantly and varies greatly because of different definitions and unstandardized methodology, but in general prevalence has been estimated to range from 3.6 to 38.4% (see Ward et al., 2012, for a recent review).

Previous neurophysiological and neuropsychological studies have concluded that, in addition to memory impairment and in comparison with age-matched controls, AAMI older adults may show a significant decline in attentional resource capacity and executive functions associated with frontal lobe function (Hänninen et al., 1997; Anderer et al., 2003). To our knowledge, electrophysiological studies trying to elucidate the mechanisms that underlie visual selective attention processes in AAMI are inexistent. Nevertheless, this is an interesting point taking into account that the ability to suppress distracting information or resist interference, a critical aspect of selective attention mechanisms, plays an important role in a broad range of cognitive functions including working memory (Gazzaley et al., 2005; Hasher, 2007; Solesio-Jofre et al., 2011, 2012).

In laboratory studies, inhibitory mechanisms of selective attention may be inferred from visual search paradigms that require subjects to search for a predefined target stimulus in arrays containing a variable number of distractor stimuli, and that may reveal deficits of filtering out or suppressing irrelevant distractors during target processing (Luck and Hillyard, 1995; Luck and Ford, 1998).

The widely known age-related deficit in visual search processes has been attributed to a decline in the inhibition of irrelevant stimuli (i.e., the inhibitory deficit hypothesis, Hasher and Zacks, 1988; Colcombe et al., 2003; Madden and Whiting, 2004). In the present study, we specifically explored whether a salient but task-irrelevant color stimulus automatically captures attention in AAMI patients. To this end, we employed a visual search paradigm in which the participants searched for a target defined by its orientation, and an irrelevant color stimulus was presented without prior announcement in separate trials. The task-irrelevant stimulus was a red bar, since red stimuli have shown to be highly salient and to attract attention more efficiently than stimuli of other colors (Pomerleau et al., 2014). Taking into account that the lateralized N2pc event-related brain potentials (ERPs) component has been considered as an index of attentional selection/filtering processes (Luck and Hillyard, 1994a,b; Eimer, 1996; Girelli and Luck, 1997; Luck et al., 1997; Hickey et al., 2009), its appearance was used as a mean to explore whether visuospatial attention is captured by the irrelevant stimulus. In this line, previous studies have reported that the presence of a salient irrelevant stimulus can automatically (bottom-up) attract attention to its spatial location (Theeuwes, 1991, 1992, 1994; Theeuwes et al., 2000; Hickey et al., 2006), significantly slowing performance if the salient stimulus is a distractor and speeding performance if is the target (see Ruz and Lupiáñez, 2002, for a review). In a previous electrophysiological study, we found that healthy older adults were not more susceptible to attentional capture than younger adults, being able to ignore the interference of task-irrelevant color stimuli (Lorenzo-López et al., 2008). With the present study we will extend our previous results using an AAMI older adults group on the same experimental task.

Prolonged N2pc latencies and decreased amplitudes have been previously reported in healthy older adults in visual search tasks (Lorenzo-López et al., 2008, 2011; Amenedo et al., 2012; Li et al., 2013; Störmer et al., 2013), indicating a decline in the correlates of allocation of attentional resources to a lateralized target stimulus. N2pc amplitude has also been shown to be smaller in patients with multiple-domain-amnestic mild cognitive impairment (MCI; Cespón et al., 2013a, 2015) than in healthy older adults. Importantly, recent promising evidence has suggested that cognitive training can counteract the age-related decline in visual selective attention, increasing the N2pc amplitude after 10 weeks of a speed of processing training conducted twice a week (O’Brien et al., 2013).

In order to study the allocation of attentional resources to a relevant target in AAMI patients, we will also explore the time course and amplitude of the N2pc component to target arrays. This is a relevant point since, to our knowledge, modulations of the specific early visuospatial attention process indexed by this component have not been studied before in these patients.

## Materials and Methods

### Participants

Event-related brain potentials were recorded from 10 AAMI older adults (four females; mean age 83.10 ± 9.80 years, age range 64–96 years), recruited from the Gerontological Complex La Milagrosa in A Coruña (Spain). All had normal or corrected-to-normal visual acuity, and reported normal color vision. They underwent a standardized diagnostic assessment comprising medical history and neuropsychological assessment to examine mental capacity and to rule out the presence of dementia, which includes the Mini-Mental State Examination (MMSE; Folstein et al., 1975; Blesa et al., 2001) and the Geriatric Depression Scale (GDS; Sheikh and Yesavage, 1986). Participants were considered to have AAMI if they met all the following diagnostic criteria (Crook et al., 1986): (1) Subjective memory complaints affecting routine activities. We specifically assessed subjective memory complaints using one sample question “Do you suffer from forgetfulness?”, (2) Objective evidence of memory loss (a score of at least 1 SD below the mean for younger adults on the Logical Memory subtest from Wechsler Memory Scale (Wechsler, 1945), (3) Absence of intellectual dysfunction, (4) Absence of dementia or any other neurological disease that affects memory (e.g., stroke, depression). Specifically, participants showed absence of dementia as determined by a score above 24 on the MMSE (mean score: 28.30 ± 1.95), and absence of depressive symptoms as determined by a mean score of 2.90 ± 2.38 on the GDS (cut point of 5 or more), (5) No medical disorders that could produce cognitive deterioration (e.g., serious cardiac disease, poorly controlled diabetes mellitus, and cancer not in remission for 2 years or longer). These ERPs were compared with those obtained in 22 healthy older adults (11 females, mean age 68.5 ± 6.0 years, age range 60–84 years), not meeting the AAMI criteria, from our previous study (Lorenzo-López et al., 2008). All participants in this control group were healthy well-functioning adults without a history of neurological or psychiatric disorder, had normal or corrected-to-normal visual acuity, and reported normal color vision.

Written informed consent was obtained from all participants prior to their inclusion in the study and their rights were protected. The experimental protocol and procedures were approved by the ethics committee of the University of A Coruña and conformed to the principles embodied in the Declaration of Helsinki.

### Stimuli and Procedure

**FIGURE 1:**
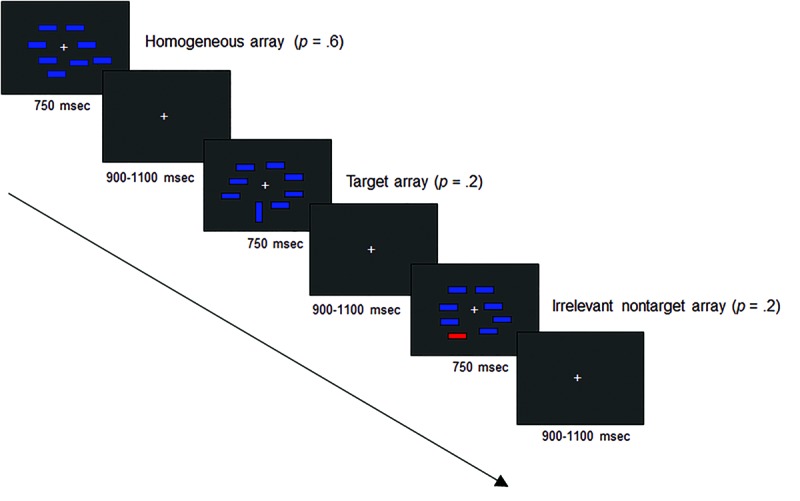
**Schematic diagram of the visual search arrays used in the event-related brain potential (ERP) experiment.** The arrow represents the time passage.

The electroencephalographic (EEG) recordings were made in an electrically shielded and sound attenuated room. Participants sat in a comfortable chair at 100 cm viewing distance from a computer display with a black background and a continuously visible white fixation cross. They were instructed to maintain central fixation and to minimize blinking while searching for a target object in arrays containing non-target distractor objects (we used the same experimental paradigm as Lorenzo-López et al., 2008, which was based on Luck and Hillyard, 1994a). The stimuli used in the experiment are shown in **Figure 1**.

Bilateral multi-element visual search arrays were presented composed of eight colored bars subtending 0.3° × 0.9° visual angle. The bars were placed at random locations within a 9.2° × 6.9° imaginary rectangular region that was centered on fixation cross. There were three types of search arrays that were randomly presented: target arrays comprising one blue-vertical bar and seven blue-horizontal bars (*p* = 0.2); non-target arrays comprising one red-horizontal bar and seven blue-horizontal bars (*p* = 0.2); and homogeneous arrays comprising eight blue-horizontal bars (*p* = 0.6). The location of target and non-target stimuli was unpredictable, and they were equally likely to appear in the right or left visual hemifields. After the fixation cross was presented for 900–1100 ms, each search array was presented for 750 ms (see **Figure 1**). The target was defined by the same feature (orientation) across all trials and the participants were not informed about the appearance of the irrelevant color distractor. Stimuli and search arrays were created, presented, and controlled using the Presentation software application (Neurobehavioral Systems, Inc., version 0.76). The experimental session was divided into six blocks of trials. Each block consisted of at least 10 orientation target arrays and at least 10 color non-target arrays presented to each hemifield, and at least 80 homogeneous arrays, to a maximum of 250 arrays in total. The task was to indicate as rapidly and accurately as possible whether the target stimulus was present or absent in each search array, by pressing a button with one hand for target-present trials and another button with the other hand for target-absent trials. Response buttons were counterbalanced across participants.

### ERP Methodology

Electroencephalography was continuously recorded using BrainAmp amplifiers (Brain Vision recorder software, Brain Products) with a 32-channel electro-cap (ECI Inc; 10–20 International System). All the active electrodes were referred to the nose tip and grounded with an electrode placed at nasion. Vertical and horizontal EOG activities were recorded bipolarly from above and below the left eye and from the outer canthi of both eyes. Electrode impedances were kept below 10 kΩ. The EEG signals were amplified (10 K) and digitized at a rate of 500 Hz/channel, and filtered on-line with a band pass of 0.05–100 Hz.

### Data Analyses

Reaction times (RTs) were recorded for the three types of search arrays. Only RTs associated with correct responses were included in the statistical analyses. Accuracy was measured as the percentage of correct responses (hit rates) to orientation targets with responses no longer than 1100 ms. A mixed design analysis of variance (ANOVA) was performed to analyze mean correct RTs, considering the *Search Array* (target, non-target, homogeneous) as the within-subject factor and *Group* (healthy controls, AAMI patients) as the between-subject factor. A one-way ANOVA with *Group* (healthy controls, AAMI patients) as the between-subjects factor was performed to analyze hit rates.

Electroencephalographic data were analyzed using BrainVision Analyzer 2.0 software (Brain Products). First, a notch filter (>50 Hz) was applied to the data. EEG was averaged off-line for epochs of 500 ms post-stimulus and 100 ms pre-stimulus. Epochs exceeding ±100 μV and those containing blinks, and horizontal or vertical eye movements were excluded from averaging, as well as epochs associated with incorrect or no responses. EEG was averaged separately for target and non-target pop-outs occurring in the right visual field (RVF) and in the left visual field (LVF) and for homogeneous arrays, resulting in five waveforms for each participant. After trial rejection, the mean number of averaged free-artifact epochs for each experimental condition was 89.3 ± 17.9 for RVF target arrays (range 68–117), 89.4 ± 7.1 for LVF target arrays (range 82–102), 61.9 ± 11.8 for RVF non-targets arrays (range 45–84), and 61.2 ± 12.3 for LVF non-targets arrays (range 44–90). As a whole, the average number of epochs analyzed was 75.4 ± 8.5, ranging from 66 to 99.5. Mean amplitude values of N2 posterior component were measured at P3/P4, PO3/PO4, O1/O2, and T5/T6 sites in each patient from 250 to 410 ms (this time window was defined based on visual inspection of ERP grand-averaged waveforms). These data were entered into an initial overall model ANOVA in which *Array Type* (target, non-target), *Electrode Location* (parietal, parieto-occipital, occipital, temporal), *Hemisphere* (left or right hemisphere electrode site), and *Laterality* (ipsilateral or contralateral relative to the electrode location) were entered as within-subject factors, and *Group* (healthy controls, AAMI patients) as the between-subjects factor. Separate ANOVAs were also performed for each search array type, with *Electrode Location, Hemisphere, and Laterality* as the within-subject factors, and *Group* as the between-subjects factor.

To isolate N2pc, difference waveforms were constructed by subtracting the ERPs for ipsilateral (relative to the electrode location) target arrays from those for contralateral target arrays. N2pc latencies and mean amplitudes were quantified in these contralateral-minus-ipsilateral waveforms from the largest negative peak and mean voltage between 250 and 410 ms after the target array onset in the AAMI participants, and between 205 and 375 ms in the healthy controls (see Lorenzo-López et al., 2008). The resulting N2pc amplitude and latency values were entered into mixed model ANOVAs with *Electrode Location* (parietal, parieto-occipital, occipital, temporal) as the within-subject factor, and *Group* (healthy controls, AAMI patients) as the between-subjects factor. Voltage maps were also computed from the difference waveforms at the time point of the maximum N2pc peak indicated by the ANOVAs, to examine potential changes in the N2pc scalp distribution between the groups. Whenever appropriate, degrees of freedom were corrected by the conservative Greenhouse–Geisser estimate. When necessary, *post hoc* comparisons were performed using the Bonferroni adjustment for multiple comparisons. An alpha level of 0.05 was used for all statistical tests. Effect sizes for ANOVAs are reported as partial eta-squared ($\eta_{p}^{2}$).

## Results

### Behavioral Results

Mean RTs in healthy older adults and AAMI patients are summarized in **Table 1** as a function of array type. AAMI participants showed a lower performance level on the visual search task [*F*(1,30) = 32.695, *p* < 0.0001; healthy older mean hit rates: 91.2 ± 12.5%; AAMI: 64.5 ± 11.6%], and slower RTs [*F*(1,30) = 7.496, *p* < 0.010, $\eta_{p}^{2}$ = 0.200; healthy older: 600.5 ± 90.3 ms; AAMI: 683.3 ± 44.1 ms]. Importantly, there was a significant *Array Type* by *Group* interaction on the RTs [*F*(2,60) = 5.240, 𝜀 = 0.587, *p* < 0.023, $\eta_{p}^{2}$ = 0.149]. As can be seen in **Table 1**, RTs were slowest for the orientation targets, intermediate for the color non-targets, and fastest for the homogeneous arrays [*Array Type*: *F*(2,42) = 16.036, 𝜀 = 0.554, *p* < 0.0001, $\eta_{p}^{2}$ = 0.433; with *p* < 0.05 for all the *post hoc* pairwise comparisons among array types] in healthy older controls. However, AAMI patients did not show significant RT differences among the three types of arrays [*Array Type*: *F*(2,18) = 0.325, 𝜀 = 0.643, *p* = 0.635, $\eta_{p}^{2}$ = 0.035].

**Table 1:** Mean reaction times (RTs, ms) and their corresponding standard deviations as a function of array type in healthy older adults and AAMI patients.

	RTs	
Array Type	Healthy controls	AAMI patients
Target	628.9 ± 88.4	681.2 ± 46.9
Distractor	590.8 ± 95.8	689.9 ± 55.4
Homogeneous	581.7 ± 95.9	678.8 ± 51.9

### ERPs Results

In the overall ANOVA where target and irrelevant non-target arrays were included, a significant *Electrode Location* by *Array Type* interaction [*F*(3,90) = 7.049, *p* < 0.002, 𝜀 = 0.693, $\eta_{p}^{2}$ = 0.190] revealed that the distribution of voltage over the scalp was significantly different for target and non-target arrays. Subsequent ANOVAs were conducted separately for each search array type, because a significant main effect of *Laterality* [*F*(1,30) = 43.857, *p* < 0.0001, $\eta_{p}^{2}$ = 0.594], and a significant *Laterality* by *Array Type* interaction [*F*(1,30) = 31.373, *p* < 0.0001, $\eta_{p}^{2}$ = 0.511] were found.

**FIGURE 2:**
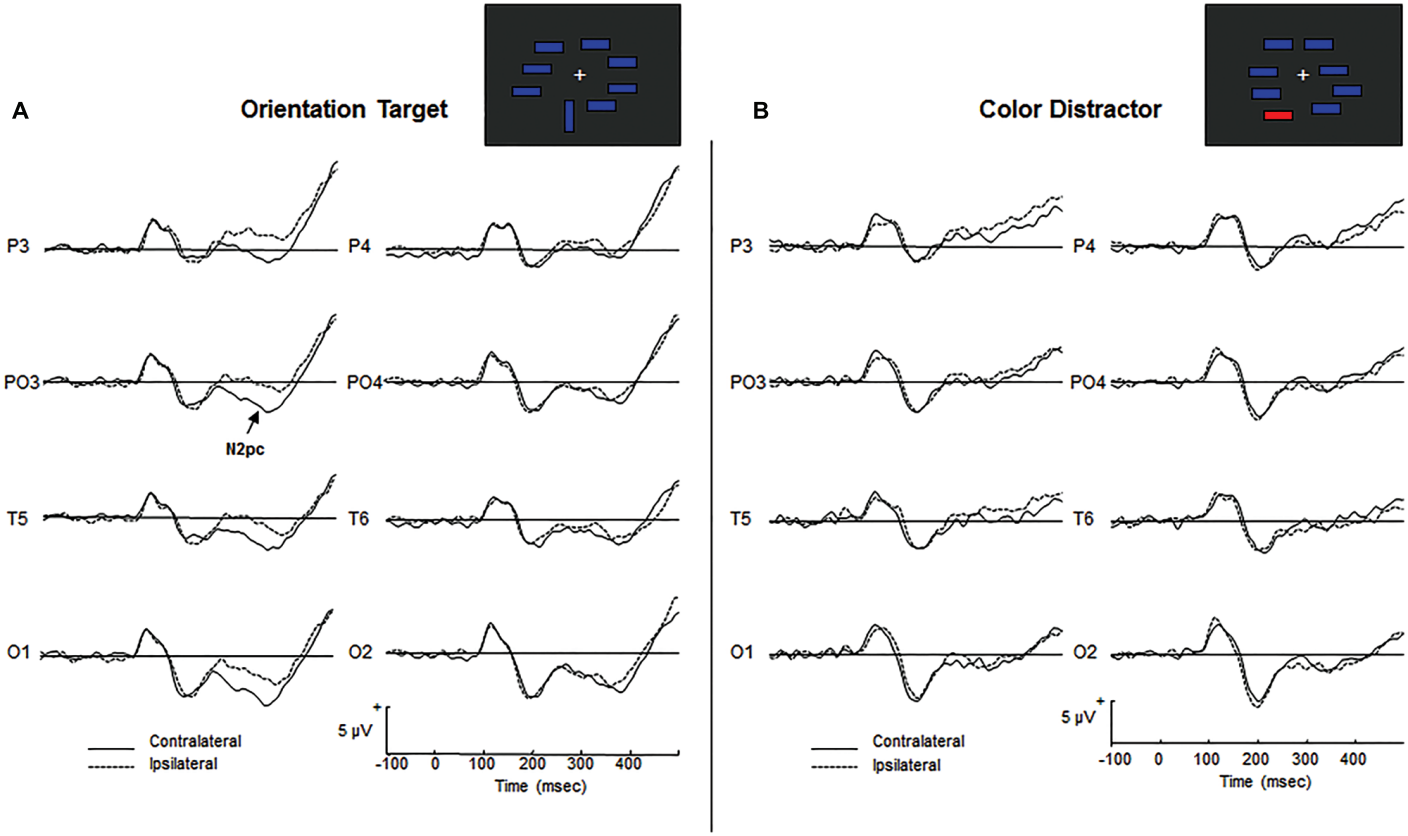
**(A)** Grand-average ERPs for targets. Superimposed are responses to targets presented in the contralateral (solid lines) and ipsilateral (dashed lines) visual field to electrode locations. **(B)** Grand-average ERPs for irrelevant non-targets. Superimposed are responses to non-targets presented in the contralateral (solid lines) or ipsilateral (dashed lines) to electrode locations. The N2pc component is absent.

For target arrays, the ANOVAs reflected the presence of the N2pc component in both groups by a significant main effect of *Laterality* [*F*(1,30) = 43.457, *p* < 0.0001, $\eta_{p}^{2}$ = 0.529]. **Figure 2A** shows the grand-average ERP waveforms for target arrays in AAMI patients, revealing a higher negativity in the N2 latency range contralateral to the target location. Visual inspection of the waveforms showed an apparent hemispheric asymmetry in its magnitude, with the amplitude difference between ipsilateral and contralateral waveforms being lower in the right hemisphere. However, the *Hemisphere* factor and the *Hemisphere* by *Laterality* interaction were not significant [*Hemisphere*: *F*(1,30) = 2.935, *p* = 0.097, $\eta_{p}^{2}$ = 0.089; *Hemisphere* by *Laterality*: *F*(1,30) = 1.010, *p* = 0.323, $\eta_{p}^{2}$ = 0.033], suggesting that the N2pc component was present in all the bilateral posterior scalp electrodes analyzed.

**FIGURE 3:**
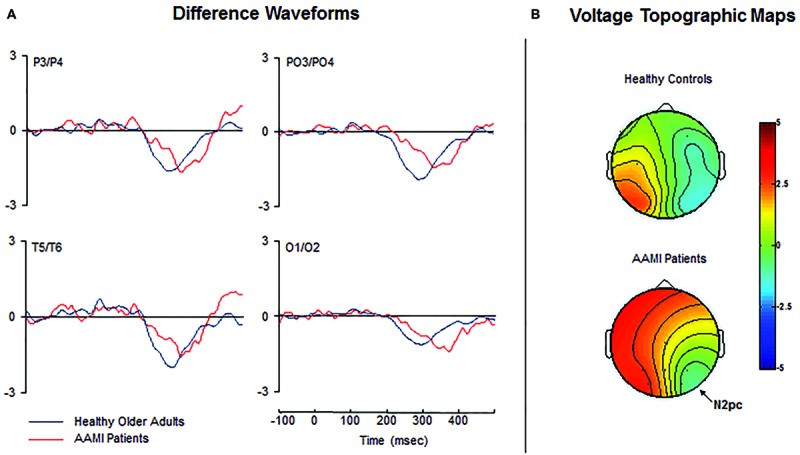
**(A)** Grand-average contralateral-minus-ipsilateral difference waveforms for healthy older adults (blue lines) and AAMI patients (red lines). **(B)** Voltage topographic maps showing the N2pc amplitude distribution in healthy older adults and AAMI patients at the latency of the peak amplitude. The N2pc topographical scalp distribution was maximal at occipital sites in both groups.

Difference waveforms are shown in **Figure 3A**. The N2pc latency was significantly delayed [*F*(1,30) = 19.979, *p* < 0.0001, $\eta_{p}^{2}$ = 0.400] in AAMI patients (347.7 ± 20.4 ms) compared to healthy older adults (308.3 ± 24.1 ms). However, mean N2pc amplitude did not significantly differ between the groups [*F*(1,30) = 0.446, *p* = 0.509, $\eta_{p}^{2}$ = 0.015; healthy controls: –0.9 ± 0.6 μV; AAMI patients: –1.0 ± 0.7 μV]. Topographical voltage maps of the contralateral-minus-ipsilateral difference waveforms are shown in **Figure 3B**, revealing similar N2pc scalp distribution over lateral visual cortical areas in both groups.

For non-target arrays, the ANOVAs revealed the absence of the N2pc component in both groups [*Laterality*: *F*(1,30) = 0.655, *p* = 0.425, $\eta_{p}^{2}$ = 0.021]. **Figure 2B** illustrates grand-average ERP waveforms for non-target arrays, showing no differences in mean amplitude between the ipsilateral and contralateral waveforms in the N2 latency range. This result suggests that the color irrelevant stimuli did not produce an automatic orienting of attention to their location in AAMI patients.

## Discussion

One of the hypotheses accounting for the normal changes in working memory processes in elderly is based on inefficient inhibitory mechanisms and diminished attentional capacity (Levitt et al., 2006). According to this point of view, inhibitory processes would significantly decline with age, so older adults would be less able to ignore or maintain out of working memory salient irrelevant information, becoming more distractible (Solesio-Jofre et al., 2011, 2012), or susceptible to attentional capture (Kramer et al., 2000). Thus, the literature provides evidence of an age-related decline in the ability to maintain an inhibitory set (Kramer et al., 2000; Colcombe et al., 2003; Greenwood and Parasuraman, 2004). In order to explore the distractor suppression mechanisms in AAMI, describing a non-disease aging decline in memory showing attentional problems, ERPs were recorded while AAMI participants searched for a simple target defined by an orientation difference to the surrounding distractors. To specifically explore the potential capture of attention to distractor locations, a task-irrelevant stimulus defined by a different unique feature (color) was also presented without their knowledge. In order to detect the possible attentional capture by these irrelevant color stimuli, analysis were focused on the N2pc ERP component reflecting brain activity specifically related to the allocation of visuospatial attention (Luck and Hillyard, 1994a,b; Woodman and Luck, 1999, 2003), and that has been previously used as a valid tool to investigate the ability of irrelevant salient stimuli to capture attention (Girelli and Luck, 1997; Eimer and Kiss, 2006; Hickey et al., 2006; Lorenzo-López et al., 2008; Rodríguez-Holguín et al., 2009; Burra and Kerzel, 2013).

Behavioral results showed a significant delay in mean RTs for both relevant target selection and successful distractor rejection, and a significant decrement in performance in AAMI patients compared to healthy controls. A reduced task-related attentional selection has been also previously reported in amnestic MCI and AD patients (Redel et al., 2012). The fact that the AAMI patients did not show RT differences between the three search array types suggests that there are differences in the processing of distractors between the groups. In the present paper, no EEG sign of capture by the irrelevant non-targets at the level of visuospatial attention (reflected by the absence of the N2pc component) was observed regardless of group, indicating a preserved top-down suppression of irrelevant information. Thus, the observed slowing in distractor rejection in the AAMI group may be explained by differences in the time course of response-related processes (selection of the appropriate motor response, and execution of the required response) but not by delays in the distractor suppression process reflected by N2pc. In this regard, potential changes in motor-related ERP activity between AAMI patients and healthy older controls should be explored in future studies with large samples. Previous studies have reported age-related deficits in motor-response production in visual search (Amenedo et al., 2012; Wiegand et al., 2013) and Simon (Van der Lubbe and Verleger, 2002; Cespón et al., 2013b) tasks.

From an electrophysiological point of view, a robust N2pc component was observed when the AAMI participants were presented with orientation target stimuli. Visual inspection of the waveforms showed an apparent hemispheric asymmetry in the magnitude of the N2pc component, which seems lower in the electrodes placed on the right hemisphere. A similar effect was observed in healthy older adults (see **Figure 1**, right panel in Lorenzo-López et al., 2008). It is important to note that spatial selective attention is indeed right hemispheric dominant (Mesulam, 1981; Shulman et al., 2010). In this regard, in a recent magnetoencephalographic study (Lorenzo-López et al., 2011) we observed a significant age-related hypoactivation of the occipito-temporal sources of the magnetic counterpart of the N2pc component (mN2pc) that was more pronounced in the right hemisphere and that could partly explain the observed hemispheric asymmetry.

Importantly, although a clear N2pc was observed in both groups for target arrays, its peak latency was significantly delayed in AAMI patients (on average 39.4 ms longer) compared to healthy controls, suggesting that they require more time to shift their visuospatial attention onto the relevant target. This slowing in N2pc latency may contribute to the general slowing observed in RTs to targets in these patients. However, no significant differences between the groups in N2pc mean amplitude and scalp distribution were found, suggesting that the amount of attention that is allocated to the target did not differ. The differential time course of the N2pc between the groups suggests that the AAMI condition involves a significant slowing of the allocation of visuospatial attention, besides their typical subjective sense of gradual memory decline in everyday situations. However, this finding must be interpreted with caution because, given the high difference in the mean age between the groups, the observed delay in RTs and N2pc latency might partially correspond to the expected latency shifts with advancing age. In this way, our results could not be specifically attributed to the AAMI process, and suggest that the previously reported age-related impairment in the allocation of visuospatial attention (Lorenzo-López et al., 2008, 2011; Amenedo et al., 2012; Wiegand et al., 2013) is clearly exacerbated as the age advances. It is important to note, however, that if the difference in age between the groups completely explains the results, a delay in N2pc latency and a reduction in its amplitude (related to slower and less effective allocation of attentional resources to the processing of the target stimulus, respectively) should be expected in the AAMI patients, which were older. However, taking into account that a non-linear relationship between N2pc peak latency and age has been recently demonstrated (Cespón et al., 2013b), and that no differences were found in the N2pc amplitude, we consider that the healthy older group was valid as a control for the present manipulation.

As previously stated, the N2pc component was absent for task-irrelevant distractors in both groups, suggesting that the color stimulus did not automatically capture the attention of participants when it was completely irrelevant for the task. In the same line, some studies have found that irrelevant stimuli in visual search do not capture attention (Leber and Egeth, 2006; Wykowska and Schubo, 2011), and that older adults demonstrate the same resistance to capture by irrelevant color stimuli than younger adults (Lien et al., 2011). We failed to find evidence of attentional capture by color in both AAMI and healthy older adults.

Due to the fact that the color irrelevant stimulus was never the target in our study and that target and distractor features remained constant across trials and were perceptually easily distinguishable (i.e., they differed only in color), it is likely that older adults (both healthy and AAMI) efficiently ignore or suppress the color dimension adopting a specific attentional set to search for the relevant dimension (orientation). In this line, it has been recently demonstrated that when the target was predictable, the N2pc to salient color distracters was abolished (Burra and Kerzel, 2013). Our results suggest that attentional capture by a simple feature is not purely stimulus-driven and can be modulated or suppressed by the top-down knowledge of the observers (Bacon and Egeth, 1994; Folk and Remington, 1998; Theeuwes and Burger, 1998; Peterson and Kramer, 2001; Connor et al., 2004; Rodríguez-Holguín et al., 2009; Hilimire and Corballis, 2014). Thus, it is possible that preserved voluntary goal-directed (top-down) mechanisms or search strategies enabled AAMI subjects to efficiently suppress processing of distracting information in the present study, avoiding attentional capture. It is also possible that given that color feature was completely irrelevant across the whole task and that participants had prior knowledge of task-relevant target features, non-targets do not require active processing to be rejected in the present experimental task (Luck and Hillyard, 1994b; Rodríguez-Holguín et al., 2009). In order to further explore the potential attentional capture by irrelevant stimuli in AAMI patients, future studies should explore other ERP components (as contralateral positivities) reflecting distractor-related processes.

A limitation of the present study is that target and task-irrelevant stimuli were presented successively making difficult generalizations to real-world perception of natural visual scenes, in which simultaneous presentation is common, causing considerably more interference than in laboratory visual search experiments. Admittedly, it cannot be completely ruled out that age differences between the groups had an influence in the present findings. Future studies with age-matched groups are needed to establish reliable conclusions.

In summary, our results provide evidence for declined visuospatial abilities in AAMI patients, who showed a reduced speed of selective attentional shifts to the task-relevant target stimulus and prolonged RTs regardless of search array. Furthermore, our findings suggest that the mechanisms of top-down suppression of irrelevant color non-target stimuli are preserved for AAMI older adults in a simple visual search task, since they were able to successfully inhibit automatic shifting of attention to their location in the visual scene. Similar interference resistance in healthy and AAMI older adults suggest preserved inhibitory function in normal aging, at least in the context of simple feature visual search, with easily distinguishable target and irrelevant non-target features. The possibility that AAMI individuals demonstrate difficult with top-down suppression under more demanding and interfering conditions or with high-valued stimuli should be explored in future studies.

## Author Contributions

All authors meet all four criteria for authorship recommended by the International Committee of Medical Journal Editors. LL-L and JC contributed to the conception and design of the work, analysis, and interpretation. LL-L, AB, and CL contributed to the data acquisition. All authors collaborated in the interpretation of data for the work. LL-L drafted the article. All authors participated in revising it critically and gave final approval of the version to be published.

## Conflict of Interest Statement

The authors declare that the research was conducted in the absence of any commercial or financial relationships that could be construed as a potential conflict of interest.
